# From Drama-Based Interventions to Public Performance: Effects on Self-Esteem and Self-Stigma in Individuals with Mental Illness

**DOI:** 10.1192/j.eurpsy.2026.11705

**Published:** 2026-07-31

**Authors:** E. Stratou, G. N. Porfyri, A. Gamvroula

**Affiliations:** 1 Department of Psychiatry, General Hospital of Argolida, Argos; 2 Department of Occupational Therapy, University of West Attica, Athens, Greece

## Abstract

**Introduction:**

Self-stigma and low self-esteem are common in individuals with psychiatric disorders, adversely affecting their social functioning and recovery. Drama-based interventions have been proposed as an empowering approach to reduce stigma and improve psychosocial outcomes.

**Objectives:**

To evaluate the effect of a structured drama-based intervention on self-esteem and internalized stigma in community-dwelling individuals with psychiatric disorders.

**Methods:**

Fifteen participants with psychiatric diagnoses engaged in 24 weekly drama-based sessions (90–120 minutes each), implemented by the department’s occupational therapist in collaboration with a volunteer drama specialist at the Psychiatric Department of the General Hospital of Argos. Self-esteem and stigma were assessed pre- and post-intervention using the Rosenberg Self-Esteem Scale (RSES) and the Internalized Stigma of Mental Illness (ISMI) scale, respectively. Paired t-tests examined pre-post differences, and Cohen’s d quantified effect size. Significance was set at p < 0.05.

**Results:**

The sample consisted of 15 individuals with psychiatric disorders, primarily male (53.3%) and aged between 41 and 59 years (66.7%). The most commonly reported diagnosis was schizophrenia (F20.0, 40%), with the majority being unmarried and unemployed. All participants were on medication and under regular psychiatric follow-up. A significant reduction was observed in the ISMI *Stigma Resistance* subscale (p = .010; d = 0.51). Although not statistically significant, additional positive trends were noted in the other ISMI subscales (Alienation, Stereotype Endorsement, Discrimination Experience, Social Withdrawal), with small effect sizes (Table 1). Self-esteem increased post-intervention on the Rosenberg Self-Esteem Scale, although this did not reach statistical significance (p = .087; d = 0.29, Table 2).

**Table 1.** Descriptive Statistics for the ISMI Scale Before and After Intervention Table 1. Long description.

ISMI Subscale	Before (Mean ± SD)	After (Mean ± SD)	Mean Change (SD)	Effect Size (Cohen’s d)	p-value
Alienation	14.9 ± 5.1	13.6 ± 4.2	–1.3 ± 2.9	0.28	0.115
Stereotype Endorsement	16.3 ± 4.8	15.5 ± 4.7	–0.8 ± 3.1	0.17	0.299
Discrimination Experience	13.0 ± 3.4	12.3 ± 3.4	–0.7 ± 3.4	0.21	0.416
Social Withdrawal	15.1 ± 4.8	14.5 ± 4.9	–0.6 ± 2.7	0.12	0.358
Stigma Resistance	11.3 ± 3.2	9.9 ± 2.2	–1.4 ± 1.9	0.51	0.010

**Table 2.** Descriptive Statistics for the Rosenberg Self-Esteem Scale Before and After Intervention Table 2. Long description.

RSES (Self-Esteem)	Before (Mean ± SD)	After (Mean ± SD)	Mean Change (SD)	Effect Size (Cohen’s d)	p-value
	25.2 ± 5.0	26.6 ± 4.6	1.4 ± 2.9	0.29	0.087

**Conclusions:**

These findings suggest that drama-based interventions-particularly those involving active participation and culminating in a public theatrical performance-can effectively reduce self-stigma and enhance self-esteem among mental health patients, supporting their psychosocial rehabilitation.

**Disclosure of Interest:**

None Declared

## Long descriptions

### Table 1. Long description

The table consists of six columns: I S M I Subscale, Before (Mean plus or minus S D), After (Mean plus or minus S D), Mean Change (S D), Effect Size (Cohen’s d), and p-value.

* Alienation: Before 14.9 plus or minus 5.1; After 13.6 plus or minus 4.2; Mean Change -1.3 plus or minus 2.9; Effect Size 0.28; p-value 0.115.

* Stereotype Endorsement: Before 16.3 plus or minus 4.8; After 15.5 plus or minus 4.7; Mean Change -0.8 plus or minus 3.1; Effect Size 0.17; p-value 0.299.

* Discrimination Experience: Before 13.0 plus or minus 3.4; After 12.3 plus or minus 3.4; Mean Change -0.7 plus or minus 3.4; Effect Size 0.21; p-value 0.416.

* Social Withdrawal: Before 15.1 plus or minus 4.8; After 14.5 plus or minus 4.9; Mean Change -0.6 plus or minus 2.7; Effect Size 0.12; p-value 0.358.

* Stigma Resistance: Before 11.3 plus or minus 3.2; After 9.9 plus or minus 2.2; Mean Change -1.4 plus or minus 1.9; Effect Size 0.51; p-value 0.010.

Navigate back to Table 1..

### Table 2. Long description

The table contains six columns and one data row. The columns are labeled from left to right as follows.

* R S E S (Self-Esteem)

* Before (Mean plus or minus S D)

* After (Mean plus or minus S D)

* Mean Change (S D)

* Effect Size (Cohen’s d)

* p-value

The data row contains the following values.

* Before (Mean plus or minus S D): 25.2 plus or minus 5.0

* After (Mean plus or minus S D): 26.6 plus or minus 4.6

* Mean Change (S D): 1.4 plus or minus 2.9

* Effect Size (Cohen’s d): 0.29

* p-value: 0.087

Navigate back to Table 2..

